# Assessment of logistical support for road maintenance to manage road accidents in Vhembe district municipalities

**DOI:** 10.4102/jamba.v11i3.705

**Published:** 2019-07-04

**Authors:** Peter Bikam

**Affiliations:** 1 Department of Urban and Regional Planning, School of Environmental Sciences, University of Venda, Thohoyandou, South Africa

**Keywords:** Road Maintenance, Local Authorities, Logistics Support, Problems, Roads, Assessment, Maintenance, Manage, Logistical, Support

## Abstract

This article discusses the problems of logistical support for road maintenance to manage road accidents in Vhembe district municipalities. A budget deficit model was used to explain the level of inadequate logistics support to manage operations and maintenance of municipal roads as a preventative measure against road accident and disaster risks. A hypothetical road maintenance deficit model informed by current literature on road maintenance was used to explain how cost of road maintenance increases exponentially if initial maintenance was not undertaken when the facility was newly constructed to draw the link between road maintenance and the risk of road accidents. Inadequate logistical support to address road maintenance backlogs in Vhembe district municipalities has been on the increase over the last 10 years. Current studies show that inadequate road maintenance can lead to the development of potholes – a major cause of road accidents and damages to motor vehicles. Literature on logistics support emphasises a comprehensive approach to road maintenance to provide a balance between funding, routine maintenance, quality of materials used for maintenance, use of stipulated specifications, the required maintenance technology, innovations and employment of qualified service providers to ensure quality roads and reduction of accidents on municipal roads.

## Introduction

Extreme weather conditions if not anticipated and planned for can damage municipal road assets. During the 2000 floods in Vhembe district, roads and storm water drainages, electricity poles and urban furniture were damaged (Vhembe District Municipality 2009–2010). The Vhembe District Spatial Development Framework (2009) showed that urban arterial roads, residential access roads and storm water drainages were damaged. Inadequate contingency plans and logistics support to tackle the sudden deterioration of municipal roads, for example, in Louis Trichardt town, the R524 and the N1 road were damaged (Vhembe District Municipality 2009–2010). Extensive damage to municipal roads was because of inadequate logistical support to manage road maintenance to avoid disaster risks such as road accidents. Regular maintenance requires adequate allocation of budget for roads rehabilitation, such as filling of potholes, repairs of road shoulders, cleaning the roads and daily routine checks to reduce accidents (Statistics South Africa 2009). Logistical support for road maintenance is a challenge to Vhembe district municipalities (Vhembe District Municipality 2015).

## Literature review

The budget for roads development is provided via Municipal Infrastructure Grant (MIG). The MIG allocation is mainly for the development of new roads and not for maintenance. It is not clear why MIG allocation does not provide adequate budget for municipal road maintenance. According to Burningham and Stankevich (2005), poorly maintained roads constrain mobility significantly and raise vehicle operating costs, increase accident rates and their associated human and property costs. It also aggravates reduction in property value when potholes in residential areas are not rehabilitated. The state of municipal roads points to inadequate logistical support (Turner 2008). Any road will gradually deteriorate through the effects of traffic, loading and extreme weather conditions such as ultraviolet radiation and draught (Maina 2008).

This article analyses inadequate road maintenance on Vhembe district roads and explores how it has led to frequent road accidents and vehicle repairs (Karani 2008). The problems include damage to roads and storm water drainage facilities, extension of untarred roads, inadequate renewal and provision for the repairs of deteriorated road facilities (Ozadamar, Ekini & Kucukyazci 2004). A report by the Medical Council and UNISA (2007) showed a total of 328 091 deaths were recorded in South Africa between 2001 and 2006 and 9.5% were because of non-natural causes. Road accidents accounted for 9.3% of the road accidents (Statistics South Africa 2008).

According to a report by the National Department of Transport (2002), 50% of roads and storm water drainage were behind in terms of roads maintenance backlogs eradication (Municipal Turn Around Strategy [MTAS] 2010). The report indicated that older roads were not refurbished because of inadequate allocation of funds. In addition, Zimu and Lestric (2004) indicated that if roads are not adequately funded and supported with logistical support tools, they could run the risk of deteriorating fast.

From a budget point of view, Padayachee (2003) indicated that there are no savings from a reduction of maintenance cost if road maintenance is undertaken sparingly (South African National Road Agency Ltd [SANRAL] 2010). Other costs associated with inadequate roads maintenance are frequent repairs of vehicles caused by road accidents, loss of life because of poor road conditions and delayed access to healthcare facilities. Municipalities require proper logistical support tools to manage road accidents.

## Research background

This study assesses the level of logistical support for road maintenance to manage road accidents (DLGH 2010). In 2010, the municipalities were required to report on infrastructure maintenance and the eradication of backlogs up to 2010. Four municipalities in Vhembe district were assessed to determine compliance with the implementation of road maintenance to reduce the backlogs. The choice of four municipalities is to give examples of inadequate logistical support for road maintenance to manage road accidents. Makhado Local Municipality (LM) was used to demonstrate the level of problems of road maintenance in Limpopo province. However, the limitations of the study are related to inadequate data on types of disaster risks and roads accident with respect to road maintenance in the municipalities.

### Statement of the research problem

Postponing road maintenance in the first 5 years of road construction results in high direct and indirect costs between the 8th and 10th years and the cost is 18 times higher in the long run. If road defects are repaired promptly, the cost is usually modest in the long run. If defects are neglected, an entire road section may fail completely, requiring full reconstruction at 18 times or more the cost, on average, of maintenance costs in the long run. The South African National Road Agency Ltd (SANRAL) estimates that repair costs can rise to six times of the maintenance costs after the 10th year of neglect and to 18 times after the 10th year of neglect. The problems were corroborated by SANRAL indication that ‘to avoid escalating costs, MIG allocation may consider ring-fencing funds for road maintenance’ (SANRAL 2003).

### Aim and objectives of the research

The research shows how logistical support to enhance municipal road repairs is an important component of roads assets maintenance in municipalities. This is because road improvements bring immediate benefits to road users through improved access to hospitals, schools and markets. For these benefits to be sustained, roads improvement must be followed with logistical support to avoid the risks of accidents during extreme weather conditions. The specific objectives of this research are:

- to assess logistical support inputs to manage road maintenance
- to use a budget deficit model to explain how inadequate funding can increase the cost of road accidents
- to discuss current quality of roads and maintenance in Vhembe district to explain how extreme weather conditions can cause more damage and consequently increase vehicle repair costs and accidents
- to suggest logistical support systems for road maintenance to manage road accidents.

### Contribution to field

The assumption by Tetley et al. (2011) that maintenance costs of roads vary with road conditions, level of logistical support, traffic volume, geographic location, climate conditions, construction methods, technical equipment and other factors was considered in the analysis. When there is no logistical support in the 8th year of the lifespan of the road facility, the cost of maintenance increases. In rural municipalities such as in Vhembe district, geographic information systems (GIS) can be used to manage road maintenance.

## Methodology

The methodology unpacks the problems of inadequate logistical support for road maintenance to manage road accidents in Vhembe district municipalities. Data were obtained from the literature on road maintenance and storm water drainages. The data included those from the Department of Transport (DoT), Centre for International Research (CSIR), Vhembe Spatial Development Framework (SDF) and Integrated Development Plan (IDP) reports from Thulamela, Makhado, Musina and Mutale LMs (2014/2015). Information on the state of municipal road maintenance was extracted. The analysis focused on deaths for which the underlying cause was road accidents. Site visits and discussions with municipal officials were undertaken to obtain primary data on road maintenance and road accidents. The analysis from the literature reviews and research papers on strategies of logistics support for road maintenance was obtained via the Internet. Budget allocation for road maintenance in the four municipalities was analysed with emphasis on Vhembe district municipalities. The analysis demonstrates how inadequate logistics support for road maintenance was lacking in municipalities. Vhembe district was used as a case study area and Makhado LM was used to highlight the level of logistics support for roads and storm water maintenance. This article links inadequate logistics support for road maintenance with poor condition of roads and extreme weather conditions as part of the causes of road accidents.

## Discussions

### How is road maintenance linked with logistics support?

Although road maintenance is high on the list of important projects in the IDPs of Vhembe municipalities, logistical supports were pointed out as insufficient (Norman et al. 2007). According to Straub (2009), road maintenance should not be of secondary importance because environmental factors including flooding can cause extensive damage to roads; however, logistics support such as GIS can track damaged roads rapidly. Vhembe road accident deaths follow the same pattern, averaging 18.1 and 16.2 deaths, respectively, per 1 000 000 population from 2001 to 2006 (Limpopo Provincial Government 2009; Vhembe SDF 2012).

Road developers highlight the need to use Pavement Management Systems (PMS) and Road Asset Management System (RAMS) for logistical supports for preventive routine maintenance and treatment of roads (ADB 2012). Contrary to the use of RAMS and PMS to support the integration of routine maintenance on daily basis, *ad hoc* approaches were used (Kosana 2011). In Limpopo province, budget for roads development increased from 48% in 2003/2005 to 50% in 2006/2007 and a further increase of 9.1% from 2006/2007 to 2007/2008, and the funds made available were not adequate. Allocations for road maintenance were R1 501 501 in 2005/2006, R1 721 800 in 2006/2007 and R2 186 462 in 2007/2008, but the backlogs were not reduced (Vhembe District IDP Reports 2003–2008). The increases were in percentages and look high, but in terms of actual disbursements to build 23 087 km of roads in 2008, they were inadequate for road maintenance of all roads (Vhembe District Municipality 2010).

### Benefits of regular roads maintenance

According to Van der Walt (2008), one of the benefits of regular maintenance of roads is job creation and reduction in road accidents. Similarly, local construction materials such as stones and gravels can reduce the costs of road maintenance (Balcerac de Richecour & Heggie 1995; Ramage-Marin 2008).

One of the supports for monitoring the cleaning of road culverts and shoulders, cutting grasses can be promoted by using GIS software adapted for road maintenance. Planned road maintenance is cheaper in the long run because it can provide significant savings and can reduce road accidents and disaster risks (Harvey 2003).

### Logical support for road maintenance

Makhado, Thulamela, Musina and Mutale LMs are faced with logistical support for road maintenance to manage road accidents. Inadequate logistical support has led to the development of potholes as discussed under the following sections.

### Inadequate routine maintenance

The most frequent reason for inadequate rehabilitation of municipal road surfaces was related to share neglect of logistical support systems for road routine maintenance. Rural roads in Vhembe district were designed to carry less than five tonnes, but trucks rely on them. Heavy-duty trucks use the R524 road and municipal roads and some of them have become impracticable because of overloading and damages to the road shoulders (Vhembe District Municipality 2013). The SANRAL in a report to DoT (2002) indicated that ‘if logistical support systems are put in place to monitor the rehabilitation of roads they can reduce road accidents and cost of maintenance’. The report showed that ‘delay in road maintenance from 3 to 5 years after construction increases the cost of repairs by 6–18 times’ (DoT 2002). In the Vhembe District IDP (2013/2014) report of the four municipalities, inadequate logistical support systems contribute to poor road surfaces (DPLG 2014). Table 1 shows that only a few kilometres of road were maintained in Limpopo province in 2008 per district including those in Vhembe district.

**TABLE 1:** Kilometres of road maintained per district in Limpopo province in 2008.

Name of district	Paved in km	Gravel km	Total km	% Paved
Waterberg	2533	5956	8890	11.50
Sekhukune	460	1494	1954	2.09
Capricorn	1108	3200	4308	5.02
Mopani	1003	1849	2852	4.55
Vhembe	1298	2751	4050	6.00
Total	6402	15 250	22 054	29.16

*Source*: Limpopo SDF (2008)

In 2008, the total length of roads earmarked for maintenance was 22 054 km; however, only 6402 km of roads were paved, representing 29.16%. Gravel roads accounted for 69.14% of roads earmarked for maintenance in 2008. The share of paved roads accounted for only 6% of paved roads in the province. About 47.18% of the roads in the district were paved, leaving 52.8% unpaved. This poses real dangers to commuters because of the impractical nature of some of the roads during the rainy season. The Road Traffic Department in Makhado indicated that between 2001 and 2006, there was a 2% increase in road accidents. The MTAS report of 2010 showed that road accidents were in part because of lack of logistical support.

### Inadequate budget allocation

Inadequate allocation of funds for road maintenance was one of the major reasons for inadequate road maintenance. Little funds are made available to purchase logistical systems to monitor road repairs. According to the revised Vhembe IDP report 2014/2015, the backlogs with respect to roads maintenance were high. This explains why the South African Local Government Association (SALGA 2003) indicated that ‘if municipal budget includes logistical support for road maintenance to monitor road accidents it can lead to sustainable rehabilitation of roads.’ For example, in 2013, the MIG expenditure per municipality in Vhembe district showed that large amount of funds was allocated for road maintenance but it was not enough to reduce the backlog. In view of this, roads maintenance continued to be undertaken on an *ad hoc* basis in the district. Table 2 shows the MIG expenditure per municipality in Vhembe district municipalities in 2013.

**TABLE 2:** Municipal grant expenditure per municipality in Vhembe district in 2013.

Municipalities	Unpaved roads (km)	Paved roads (km)	Allocated (R’0000)	Transferred (R’000)	Expenditure (R’000)	Expenditure as % allocation	Expenditure as % transferred	Balance unspent (R’000)
Musina	667	415	14 604	14 604	6289	43.06	43.06	8315
Mutale	415	140	16 977	16 977	11 943	70.35	70.35	5034
Thulamela	710	355	74 355	74 355	62 771	84.42	84.42	11 584
Makhado	911	355	67 400	67 400	34 726	51.52	51.52	32 674
Vhembe DM	-	-	359 404	359 404	316 212	316 212	316 212	43 192

*Source*: Vhembe District 2011/2012 IDP report and Limpopo SDF 2013

Table 2 shows that National Treasury transfers were up to date, but the condition was that they should be used for the construction of new roads. This means that a major source of funds for roads development cannot be used for the maintenance of newly constructed roads and logistical support for road maintenance and the management of road accidents. It is expected that municipalities generate enough revenue from equitable share if not committed to other uses. However, Dèmurger (1999) indicated that municipalities are lagging behind in terms of road maintenance because of their low revenue base which is inadequate to fund road maintenance projects and the logistical support tools. The road maintenance backlogs in Vhembe district municipalities are shown in Table 3.

**TABLE 3:** Lengths of roads earmarked for maintenance per municipality in 2013.

Municipality	Unpaved roads (km)	Paved roads (km)
Musina	667	415
Mutale	415	140
Thulamela	710	355
Makhado	911	355
Total municipal roads	2708	1265
Vhembe DM roads	1298	2751

*Source*: Vhembe District IDP (2011–2012); Limpopo SDF (2013)

In 2013, unpaved roads constituted 68.16% of the total road network. The total road network, paved and unpaved, at the municipal level excluding districts roads was 3973 km. The district received MIG allocation of R359 404 in 2013 for new infrastructure development, but it was not used to purchase GIS-based road maintenance support systems (Vhembe District Municipality 2011–2012).

### Inadequate budget for logistical support

Although the National Treasury allocates funds to municipalities in Vhembe district, the funds can only be used for new construction and not for logistical support for road maintenance to manage road accidents. On logistical support, Tetley et al. (2011) indicated as follows:

Regarding the allocation of funds for routine and periodic road maintenance in South Africa, funding is insufficient to address actual needs for roads network poor conditions. (n.p.)

To ascertain if Tetley’s assumption that funds for municipal roads maintenance are insufficient, we looked at the allocations in Makhado municipality from 2004 to 2014 to determine if there is evidence of roads maintenance budget deficit. Table 4 shows the yearly average actual budget allocation versus the backlog deficit in rand over a period of 10 years from 2004 to 2014.

**TABLE 4:** Roads maintenance budget deficit in Makhado municipality 2004–2014.

Year of allocation	Length of roads to be maintained in km	Actual allocation in rand plus an annual increase assumed to be R2000.00	Annual increase of maintenance cost at R25 per km in the first 5 years	Budget deficit in rand
2004	2766	69 150	69 150	00.00
2005	2891	71 150	72 275	3125
2006	3029	73 150	75 725	3450
2007	3154	75 150	78 850	3125
2008	3290	77 150	82 250	3400
2009	3415	79 150	85 375	3125
2010	3538	81 150	88 450	3075
2011	3878	83 150	241 450	153 000
2012	3991	85 150	292 300	50 850
2013	4221	87 150	395 800	103 500
2014	4346	89 150	452 050	56 250
2015	4400	91 150	476 350	24 300

*Source*: Makhado Municipality (2004–2005).

Hypothetically, roads construction and maintenance manuals and logistical support systems can facilitate the monitoring of newly constructed roads in the first 5 years. Maintenance cost will be negligible because GIS can be used as a monitoring tool. However, if minimal maintenance is left unattended to in the first 5 years, from the 8th year the cost will be six times higher than the first 5 years and 18 times higher if initial repairs were neglected (National Treasury 2007).

**FIGURE 1:**
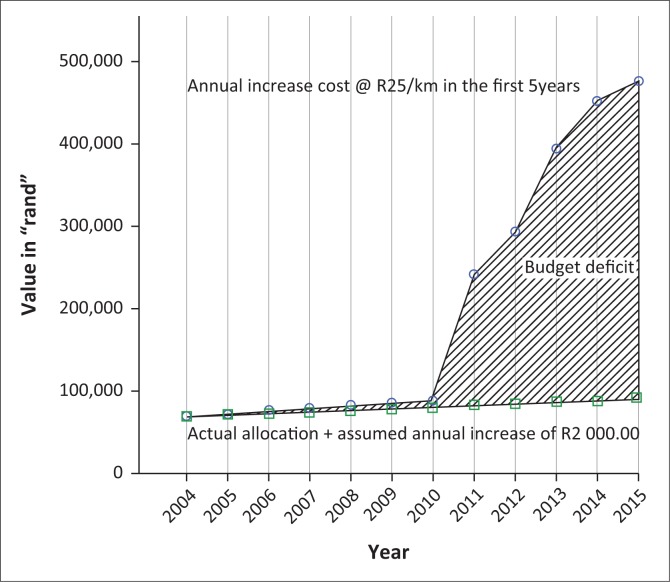
Illustration of road maintenance budget deficit model in Makhado LM 2015.

Considering Tetley’s assumptions and the figures in Table 4, if road maintenance was about R25 per km in the first 5 years, using 2004 as the base year we can plot the budget deficit model graph. At the end of the 8th to 10th years, cost increases sixfold because of wear and tear of the roads, a major cause of road accidents. If the road facility was taken care of during the first 5 years, maintenance cost would be minimal and negligible in the later lifespan of the road facility. If initial repairs were neglected for the lifespan of the road, the cost would be 18 times higher than the initial repair cost (O’Flaherty 2002). If logistical support and minor repairs were attended to, the lifespan of the road facility will be extended. As shown in Figure 1, the budget for roads maintenance in Makhado from 2004 to 2014 has declined as new roads were added. This confirms Tetley’s assumption that neglecting logistical support in the first 5 years can increase maintenance cost by the 8th to 10th years of the road facility life.

**FIGURE 2:**
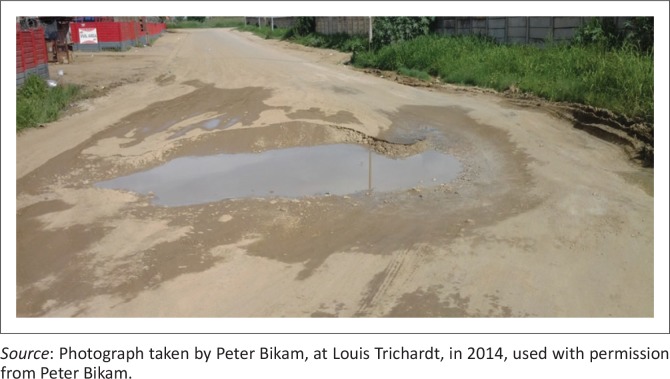
A typical road conditions in Louis Trichardt, 2014.

Figure 1 shows that budget allocation decreased but newly constructed roads increased. Municipality will have more roads to maintain if not undertaken soon after construction and logistical support systems put in place. The budget deficit gap (shaded portion) shows huge amount of deficit in the later life year of the road facility. Figure 2 is a typical example of the road conditions in Makhado LM in 2015.

Figure 2 shows an unrepaired road where water accumulated and seeped through, causing further damage to the road. The average allocation for repairs and maintenance of infrastructure in general is less than 10% of the total yearly budget (Vhembe District IDP 2013–2014).

### Poor road conditions a disaster risk

In 2015, road surfaces in Vhembe district municipalities had many potholes and crocodiles’ cracks. The characteristics of some of the road conditions are discussed in the following sections.

### Roads surface conditions in Vhembe district

The quality of a road surface in part depends on the extent to which the entire road formation is exposed to extreme weather conditions such as heavy rain, wear and tear (WHO 2006). Water weakens the base layer of the road pavement and eventually the subgrade causing more damage to the road. Therefore, it is necessary to provide logistical support systems for road maintenance to manage road accidents. The average annual rainfall in Vhembe district municipalities was 372 mm per square metre from 2004 to 2014 (Musina Weather Station South Africa 2014). One key factor in road design and construction is how to reduce the possibility of skidding when it rains. Logistical systems can measure the spacing between chipping and height to which the chipping protrudes make up the surface’s macro-texture; hence, the closer the surface chipping to one another and the higher they protrude on the road surface and friction of the tyres of passing vehicle, the greater the ability to reduce skidding (Heggie, Ian & Vickers 1998).

Municipalities do not often consider logistical support as a matter of requirement to manage roads construction, rehabilitation and surface repairs. Software can facilitate calculating when to use single seal, double seal, cape or slurry seals, stone matrix asphalt (SMA), asphalt or premix, hot-rolled, asphalt, concrete, block and brick paving, composite surface, strain alleviation membrane (SAM), stress alleviation membrane interlayer (SAMI) and ultra-thin friction course (UFTC) (Heggie, Ian & Vickers 2009). For example, defects on road surfaces, such as cracking, and joints deficiencies can be monitored with GIS based in Vhembe district municipalities. Some road surfaces showed cracks on non-grid pavements and traffic induced longitudinal cracks in the wheel path of the vehicles (Sohn et al. 2006). The cracks were also because of inferior quality construction materials. This often leads to shrinkage because materials such as cement over time can lead to exceptional wider cracks (Kevin 2007).

### Inadequate road maintenance standards

Operations and maintenance plans for roads in Thulamela, Makhado and Musina LMs were in place in 2015, but compliance to the standards was not in line with logistical support systems of norms standards. Ungazetted norms and requirements on the age of roads, categories and lengths, maintenance history and software requirements to monitor them were lacking; thus, not less than 30% of urban roads continue to deteriorate every year (Snoeren et al. 2007).

### Compliance with road maintenance legislations

It is not the absence of policies and legislation to use GIS-based logistical support to manage road maintenance, but compliance to standards (Haddow et al. 2007). The National Treasury makes allocation to municipalities, but there is little guideline on the use of GIS-based logistical support for road maintenance to manage road accidents.

The Generally Accepted Municipal Accounting Practice (GAMAP) requires municipalities to determine depreciation model of road assets by using relevant computer software which considers budgeting and monitoring expenditure for strategic maintenance, depreciation practice, defining value and issues related to roads and storm water asset characteristics and records. GAMAP requires that an asset register is drawn up for LMs.

### Inadequate roads maintenance plans

**FIGURE 3:**
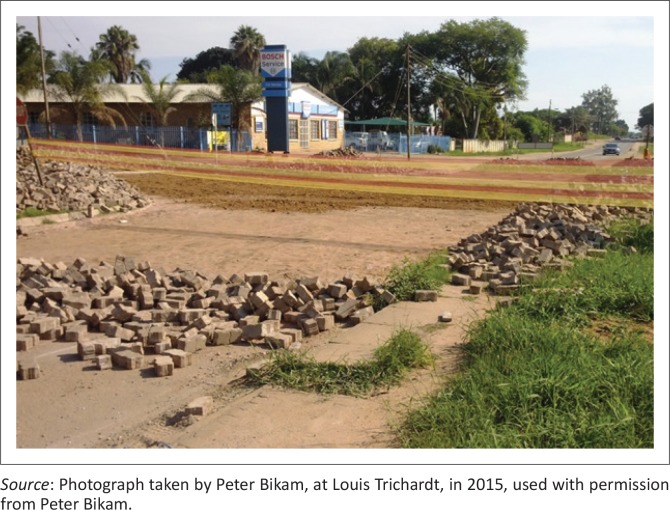
Repairs of road junctions in Louis Trichardt, 2015.

In Makhado, Thulamela and Musina municipalities, road operations and management plans were in place (IDP 2013–2014). For example, in the last 5 years, Makhado municipality grabbled with the maintenance of access roads, particularly in the residential neighbourhoods of old and new towns. During the last quarter of 2014, efforts were made to repair the potholes along Resik, Krogh and other badly damaged residential roads in Louis Trichardt and Madombidzha. Figure 3 shows the conditions of some of the road junctions in Louis Trichardt industrial area in 2015.

**FIGURE 4:**
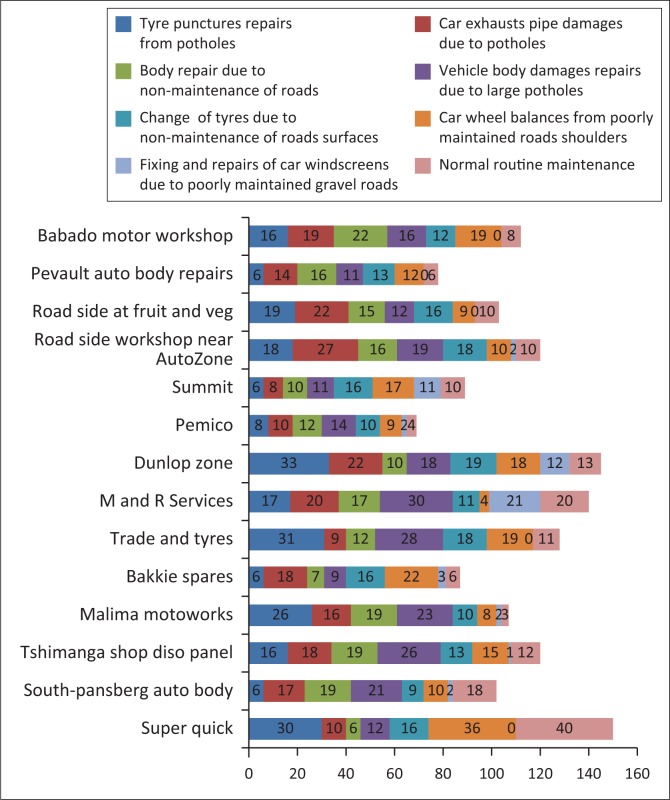
Monthly % vehicle repairs with respect to road conditions in Louis Trichardt 2015.

Figure 3 shows one of the road junctions under repair; however, it was the third time that it was repaired. The road conditions were observed to ascertain the extent to which they were linked to the frequency of motor vehicle repairs in Louis Trichardt. This was verified with the response given by motor vehicle repair workshops shown in Figure 4.

Figure 4 shows the type of repairs per motor vehicle workshop in Louis Trichardt town in 2015 per month. On average, poor road conditions accounted for not less than 70% of all repairs out of a total of 250 repairs. In 2013, the municipality embarked on road maintenance but the 2014 rains rendered the road repairs impracticable.

Municipalities do not always comply with the *Municipal Finance Management Act* (MFMA 2015–2016), Circular No. 75, Act No. 56 of 2003, with respect to providing the logistical support for roads repairs. Municipal Budget Circular for the 2015-2016 period requires municipalities to make provision for logistical support to manage road maintenance. A study by CSIR (2006) indicate that ‘the state of municipal infrastructure in South Africa and its operation and maintenance show that there is low level of infrastructure maintenance research and analysis in general’. The study showed that logistical support for road maintenance to manage road accidents was not in place. Logistics support to manage asset accounting planning and provision for funds for the maintenance of road assets is inadequate in Vhembe district municipalities. There is no compliance in terms of putting in place road maintenance policies, by-laws and logistics support (Orr 2006).

In 2014, Thulamela and Makhado LMs attempted to undertake road maintenance, without logistical support tools, and were not able to achieve a significant standard maintenance routine. The SDF reports from the four municipalities showed that road assets and the asset registers were not updated and/or reviewed regularly (Igbal, Mehler & Yildirim 2007).

### Large areas of road maintenance

The wide area of coverage means that several kilometres of roads should be constructed, maintained and upgraded every year. Similarly, demands and construction of new roads drain the funds, which could have been used for road maintenance. GIS-based road monitoring device can be used to manage large geographical area. Population is a factor in building new roads. For example, Musina municipalities had a total population of 1 294 722 people. This means that the ratio of length of roads repaired or built to the population was high (Vhembe District Municipality 2009–2010).

Irrespective of which municipality, the common denominator in the four municipalities in Vhembe district can be summarised as follows:

- Emphasis is on new roads construction and little on logistical support for road maintenance to manage road accidents.
- Little attention is given to regulatory, safety directions and signage on municipal roads.
- The assumption that road maintenance can be handled by any technician does not conform to the norms and standards required.
- Risks associated with extreme weather conditions can be monitored by GIS-based logistical support systems.

In view of the commonalities of problems faced by LMs in Vhembe district municipalities, logistical support for road maintenance to manage road accidents will enhance planed and preventative maintenance.

### Ethical consideration

This article followed all ethical standards for a research without direct contact with human or animal subjects.

### Can logistics supports enhance road maintenance?

From the preceding analysis, it is clear that the problem of road maintenance in Vhembe district was because of the piecemeal approach to the use of logistics support to manage road maintenance. The comprehensive approach to tackle the problems holistically includes the following.

### Ensure comprehensive approach to road maintenance

The municipalities require a comprehensive approach to address the problems faced by the local authorities with respect to road maintenance. The National Infrastructure Maintenance Strategy requires municipalities to adhere to the following:

- Strengthen the regulatory framework on the planning and budgeting for roads infrastructure.
- Ring-fence funds for roads infrastructure maintenance and the relevant logistical support tools.
- Municipalities should monitor road conditions through a GIS online evaluating reporting platform.
- Introduce a performance-based road maintenance for municipalities.

### Ensure strengthening regulatory framework

Municipalities should consider harmonising and strengthening the regulatory framework for planning and budgeting for logistical support to manage road accidents. The National Treasury should reconsider the criteria for the allocation of MIG to include provision of logistical support for road assets maintenance. In terms of *Government Immovable Asset Management Act* (GIAMA) of 2007, Article 14(1) and Article (21), municipalities should plan for road maintenance including the use of logistical support systems. The yearly budget meant for road maintenance as well as performance monitoring should be ring-fenced by municipalities. The municipalities should consider ensuring a comprehensive approach to road maintenance with logistical support to manage road accidents and disaster risks including the following.

#### Introduce a performance-based road maintenance contracting

The legislations on sustainable road maintenance can provide for roads construction contractors to use GIS support systems for planning, design and implementation of roads maintenance to achieve road maintenance objectives.

#### Introduce a geo-information spatial system

As almost there is one natural disaster in the form of extreme weather conditions in Vhembe district municipalities, a spatial data information system should be put in place to monitor extreme weather conditions with respect to road accident reporting. Spatial information on roads can enhance monitoring of road conditions. The geo-information technology if properly used can facilitate the integration of spatial data for making informed decision for sustainable road maintenance to avoid road accidents.

## Conclusion

This study used Vhembe district to show that lack of logistical support and inadequate provision of funds were one of the causes of road accidents and explains why there are backlogs in road infrastructure maintenance. Lack of road infrastructure maintenance and insufficient logistics support are linked. In Vhembe district, municipalities can be improved if proper planning and funding are provided. A road maintenance budget deficit model in Makhado LM was used to show hypothetically how the lack of initial maintenance of the road facility can multiply the cost of road maintenance exponentially in the 8th to 10th years. It was demonstrated that to reduce the rate of road accidents and vehicle repairs costs and disasters linked to inadequate road maintenance, logistical support problems should be addressed. The local municipalities should address the technical skills required for road infrastructure maintenance, including strengthening capacity and experience. Resources must be ring-fenced to purchase GIS-based logistical support systems for roads maintenance. Other measures should include strengthening the road maintenance regulation and governance framework to be put in place and linked to adequate budget allocation and proper investment in the software required to manage roads maintenance.
